# Apriori prediction of chemotherapy response in locally advanced breast cancer patients using CT imaging and deep learning: transformer versus transfer learning

**DOI:** 10.3389/fonc.2024.1359148

**Published:** 2024-05-02

**Authors:** Amir Moslemi, Laurentius Oscar Osapoetra, Archya Dasgupta, David Alberico, Maureen Trudeau, Sonal Gandhi, Andrea Eisen, Frances Wright, Nicole Look-Hong, Belinda Curpen, Michael C. Kolios, Gregory J. Czarnota

**Affiliations:** ^1^ Physical Sciences, Sunnybrook Research Institute, Toronto, ON, Canada; ^2^ Department of Medical Oncology, Department of Medicine, Sunnybrook Health Sciences Centre, Toronto, ON, Canada; ^3^ Department of Medicine, University of Toronto, Toronto, ON, Canada; ^4^ Department of Surgical Oncology, Department of Surgery, Sunnybrook Health Sciences Centre, Toronto, ON, Canada; ^5^ Department of Surgery, University of Toronto, Toronto, ON, Canada; ^6^ Department of Medical Imaging, Sunnybrook Health Sciences Centre, Toronto, ON, Canada; ^7^ Department of Medical Imaging, University of Toronto, Toronto, ON, Canada; ^8^ Department of Physics, Toronto Metropolitan University, Toronto, ON, Canada; ^9^ Department of Radiation Oncology, University of Toronto, Toronto, ON, Canada; ^10^ Department of Radiation Oncology, Sunnybrook Health Sciences Centre, Toronto, ON, Canada; ^11^ Department of Medical Biophysics, University of Toronto, Toronto, ON, Canada

**Keywords:** neoadjuvant chemotherapy, LABC, deep learning, ViT transformer, response prediction and CT imaging

## Abstract

**Objective:**

Neoadjuvant chemotherapy (NAC) is a key element of treatment for locally advanced breast cancer (LABC). Predicting the response to NAC for patients with Locally Advanced Breast Cancer (LABC) before treatment initiation could be beneficial to optimize therapy, ensuring the administration of effective treatments. The objective of the work here was to develop a predictive model to predict tumor response to NAC for LABC using deep learning networks and computed tomography (CT).

**Materials and methods:**

Several deep learning approaches were investigated including ViT transformer and VGG16, VGG19, ResNet-50, Res-Net-101, Res-Net-152, InceptionV3 and Xception transfer learning networks. These deep learning networks were applied on CT images to assess the response to NAC. Performance was evaluated based on balanced_accuracy, accuracy, sensitivity and specificity classification metrics. A ViT transformer was applied to utilize the attention mechanism in order to increase the weight of important part image which leads to better discrimination between classes.

**Results:**

Amongst the 117 LABC patients studied, 82 (70%) had clinical-pathological response and 35 (30%) had no response to NAC. The ViT transformer obtained the best performance range (accuracy = 71 ± 3% to accuracy = 77 ± 4%, specificity = 86 ± 6% to specificity = 76 ± 3%, sensitivity = 56 ± 4% to sensitivity = 52 ± 4%, and balanced_accuracy=69 ± 3% to balanced_accuracy=69 ± 3%) depending on the split ratio of train-data and test-data. Xception network obtained the second best results (accuracy = 72 ± 4% to accuracy = 65 ± 4, specificity = 81 ± 6% to specificity = 73 ± 3%, sensitivity = 55 ± 4% to sensitivity = 52 ± 5%, and balanced_accuracy = 66 ± 5% to balanced_accuracy = 60 ± 4%). The worst results were obtained using VGG-16 transfer learning network.

**Conclusion:**

Deep learning networks in conjunction with CT imaging are able to predict the tumor response to NAC for patients with LABC prior to start. A ViT transformer could obtain the best performance, which demonstrated the importance of attention mechanism.

## 1 Introduction

Locally advanced breast cancer (LABC) is a diverse condition that presents in various clinical forms (1, 2). It encompasses tumors that are larger than 5 cm or involve the skin and chest wall (1, 2). Additionally, LABC includes inflammatory breast cancer and cases where patients have fixed axillary lymph nodes or involvement of nodes in the ipsilateral supraclavicular, infraclavicular, or internal mammary regions (1, 2). Managing LABC remains a formidable clinical challenge since the most individuals with this stage of disease tend to have poorer survival rates compared to those with early-stage breast cancer (1, 2).

The standard approach for treating LABC involves a multimodal strategy consisting of systemic therapy, surgery, and radiotherapy (1, 2). In certain cases, the possibility of resecting inoperable tumors becomes viable, particularly with the use of Neoadjuvant chemotherapy (NAC), which helps shrink the tumors. This is followed by surgical intervention and subsequent adjuvant radiotherapy, and targeted therapy or hormonal therapy when indicated (3).

Treatment with Neoadjuvant chemotherapy (NAC) in locally advanced breast cancer (LABC) often yields variable responses, with only 15-40% of cases eventually achieving a complete pathological response to this treatment (4). It’s crucial to note that the pathological response of tumors to NAC serves as a critical prognostic indicator for long-term disease-free survival (DFS) and overall survival (OS) in specific patient groups (5, 6). However, several months after the therapy has started the conventional assessment of treatment response in LABC tumors to NAC occurs at the end of the treatment course. This evaluation typically relies on pathological assessments, often using the Miller-Payne (MP) grading system to compare tumor cellularity between pre-treatment core needle biopsies and post-treatment surgical specimens (6, 7). Given the invasive nature of these methods, there is a growing interest in non-invasive imaging techniques to evaluate therapy responses in LABC tumors. The goal is to identify imaging biomarkers that can predict tumor responses early in the course of NAC, facilitating personalized treatment strategies.

Both histopathology analysis and quantitative imaging techniques have provided insights into different characteristics that can help identify how LABC tumors respond to NAC. Responsive LABC tumors, for instance, tend to exhibit lower levels of cell proliferation compared to non-responsive tumors, often due to an increase in apoptosis (8, 9). Additionally, studies have shown a correlation between the expression of the human epidermal growth factor receptor 2 (HER2) and the response to NAC (10). HER2-positive tumors have significantly higher rates of achieving a complete pathological response compared to HER2-normal tumors (10). Prior investigations using diffuse optical spectroscopic techniques have reported significant differences in hemoglobin content changes after just one week of therapy between cases with complete pathological responses and those with incomplete responses (11–13). Furthermore, studies employing magnetic resonance imaging (MRI) (14) and measurements of circulating DNA and RNA integrity (15) have assessed response prediction shortly after the initiation of chemotherapy.

In cancer imaging, textural radiomics features are widely being used in the context of quantitative imaging (16–18). Previous studies have applied textural radiomics features for LABC therapy response prediction using different modalities (19, 20). Likewise, different imaging modalities have been utilized to extract informative information to build a predictive model to analyze the cancer treatment performance prior to start. In this regard, dynamic contract-enhanced magnetic resonance imaging (DCE-MRI) (14), positron emission tomography (PET) (21, 22), Diffuse optical imaging (DOI) (23), Ultrasound (US) imaging (24–26) and quantitative ultrasound (27–29) employed to assess the treatment response to breast cancer. Additionally, fusion of two different of modalities can be employed to obtain more discriminative features. To this end, Quantitative ultrasound Spectroscopic and CT information were fused in feature level to predict the response of head and neck cancer to radiation therapy treatment (30).

Although textural radiomics features are widely applied to evaluate the treatment of cancer, “detail” features, which are the most informative, can be extracted by deep learning-based techniques. Radiomics-based techniques are limited to extracting features at a superficial level, whereas deep learning techniques can delve deeper to extract features. To this end, a hierarchical self-attention-guided deep learning algorithm was trained to predict the chemotherapy treatment response using digital histopathological images (23). Likewise, in another study, outcome of radiotherapy for brain metastasis was predicted using the combination of deep learning features and clinical features. In this study, a deep convolutional neural network (CNN) was trained on MRI images to extract MRI features and thus deep textural MR-features are combined with clinical features to predict the outcome of treatment (31). Fujima et al. (32) conducted a study to predict treatment outcome for patients with oral cavity squamous cell carcinoma using deep learning and FDG-PET imaging.

Two types of deep learning networks have been widely employed to predict treatment outcomes using medical imaging. CNN-based techniques, which is called transfer learning, are applied to extract textural features from medical images (33). CNNs extract features using convolutional filters and reduce the dimension using pooling layer. The extracted features are more detailed in last layers. It means initial layers extract general features and the last layers extract details. The last layer of CNNs is flattened and then flatten layer is considered as an input of a fully connected layer (multi-layer perceptron).

Although these networks such as ResNet-50, ResNet-101, ResNet-152, Inception-V3 and Xception showed good performance to predict treatment outcomes, these CNN-based methods suffer the lack of attention mechanism. Nevertheless, vision transformer (ViT) is developed based on attention mechanism (self-attention) and it can increase the importance of image that carries the essential information (34).

The objective of this study is to evaluate deep learning networks to predict treatment outcomes for patient with LABC using CT imaging. We hypothesize that extracted features from CT images using deep learning techniques can provide vital information to predict response to NAC prior to start for patients with LABC.

Deep convolutional neural networks (CNNs) can be applied to classify medical images. These networks extract features using convolution filters by applying a convolutional operation on images. CNNs are translation invariance, which means if a filter learn information of object in one position of image, it does not need to learn same object in other position (33). In this study, five networks including VGG16, VGG19, ResNet-50, Res-Net-101, Res-Net-152, InceptionV3 and Xception were used to classify tumor response to NAC.

Convolutional neural networks (CNNs) work well for classification, segmentation, object detection and registration tasks (33). However, the lack of an attention mechanism to increase the weight of important parts of image (data) plays a limiting role in CNNs. Attention mechanisms were found in natural language processes (NLP) at first (35). The vision transformer (ViT) emerged to compensate for the lack of an attention mechanism in traditional CNNs (36). The attention mechanism is the backbone of ViT methodology and it improves the understanding of a global representation of data, which leads to an improvement of the learning during training phase by increasing attention of network on important information. ViT splits the images into patches and then patches are flattened to have linear sequences. Since the spatial dependency among patches is significantly important, positional encoding is performed in ViT to assign the position of each patch in embedding space.

## 2 Materials and methods

### 2.1 Study protocol and data acquisition

This research was carried out in compliance with the ethical guidelines set by Sunnybrook Health Sciences Center (SHSC) and Sunnybrook research Institute (SRI). The study included a total of 117 patients, comprised of 82 responders and 35 non-responders, who were diagnosed with locally advanced breast cancer (LABC) and undergoing neoadjuvant chemotherapy (NAC). All patients provided written informed consent. Tumor sizes were determined through MRI scans performed as part of standard care. Pre-treatment core needle biopsy specimens were subjected to histopathological analysis, confirming a cancer diagnosis for all patients. Post-operative pathology specimens provided crucial information about initial cellularity, tumor subtype, and the expression of hormone receptors, including estrogen receptor (ER), progesterone receptor (PR), and HER2 status as part of stand of care. All patients completed a full course of NAC, typically lasting 4-6 months. Following surgery, patients received adjuvant therapies in accordance with standard institutional practices, which included radiation, maintenance Trastuzumab for HER2-positive tumors, or endocrine therapy for hormonal-receptor positive tumors.

### 2.2 Pathological evaluation of tumor response

After finishing a full NAC regimen, patients underwent either lumpectomy or mastectomy. As part of their clinical care, standard clinical data and histopathological assessments of treatment outcomes were used to evaluate the pathological response of tumors to NAC. Specifically, patients were categorized into two groups: non-responders (referred to as “NR”) consisting of patients with stable disease or progressive disease and responders (referred to as “R”) consisting of patients with partial or complete response. This classification was determined using a modified response (MR) grading system, which drew from the Response Evaluation Criteria in Solid Tumor (RECIST) (37) and residual tumor cellularity (6). RECIST assesses the percentage change in tumor size (measured in its longest dimension) before and after treatment. A MR score of 1 indicates that there was no decrease in tumor size. MR score of 2 corresponds to a reduction in tumor size of up to 30%. An MR score of 3 is linked to a reduction in tumor size ranging from 30% to 90%. An MR score of 4 is indicative of a reduction in tumor size exceeding 90%. An MR score of 5 signifies the absence of any remaining evidence of a tumor.

In addition, to these criteria based on RECIST measurements, we also took into account the residual tumor cellularity to evaluate the treatment response. Specifically, we established a threshold of 5% for tumor cellularity. Patients are categorized as responders if tumors have cellularity equal to or less than 5% ( ≤ 5%), otherwise they are categorized as non-responders. There was no case with cellularity equal to or less than 5% prior to start.

Overall response assessment integrated both the RECIST-based criteria concerning tumor size reduction and the assessment of residual tumor cellularity. According to the RECIST criterion, a patient was classified as a responder (‘R’) if either there was a reduction in tumor size exceeding 30% (MR score 3-5) or if the residual tumor cellularity was low (<=5%). Conversely, a patient was categorized as a non-responder (‘NR’) if the reduction in tumor size was less than 30% (MR score 1-2) or if there was an increase in tumor size residual tumor cellularity was high (>5%).

The RECIST-based criteria and the evaluation of residual tumor cellularity were used to determine the target response for binary classification.

### 2.3 Data pre-processing and deep learning

Oncologists characterized the regions of interest (ROI) for all CT image slices throughout the whole tumor. Transformer and transfer-learning techniques as deep learning approaches were considered to discriminate responder from non-responder patients.

**Figure 1:**
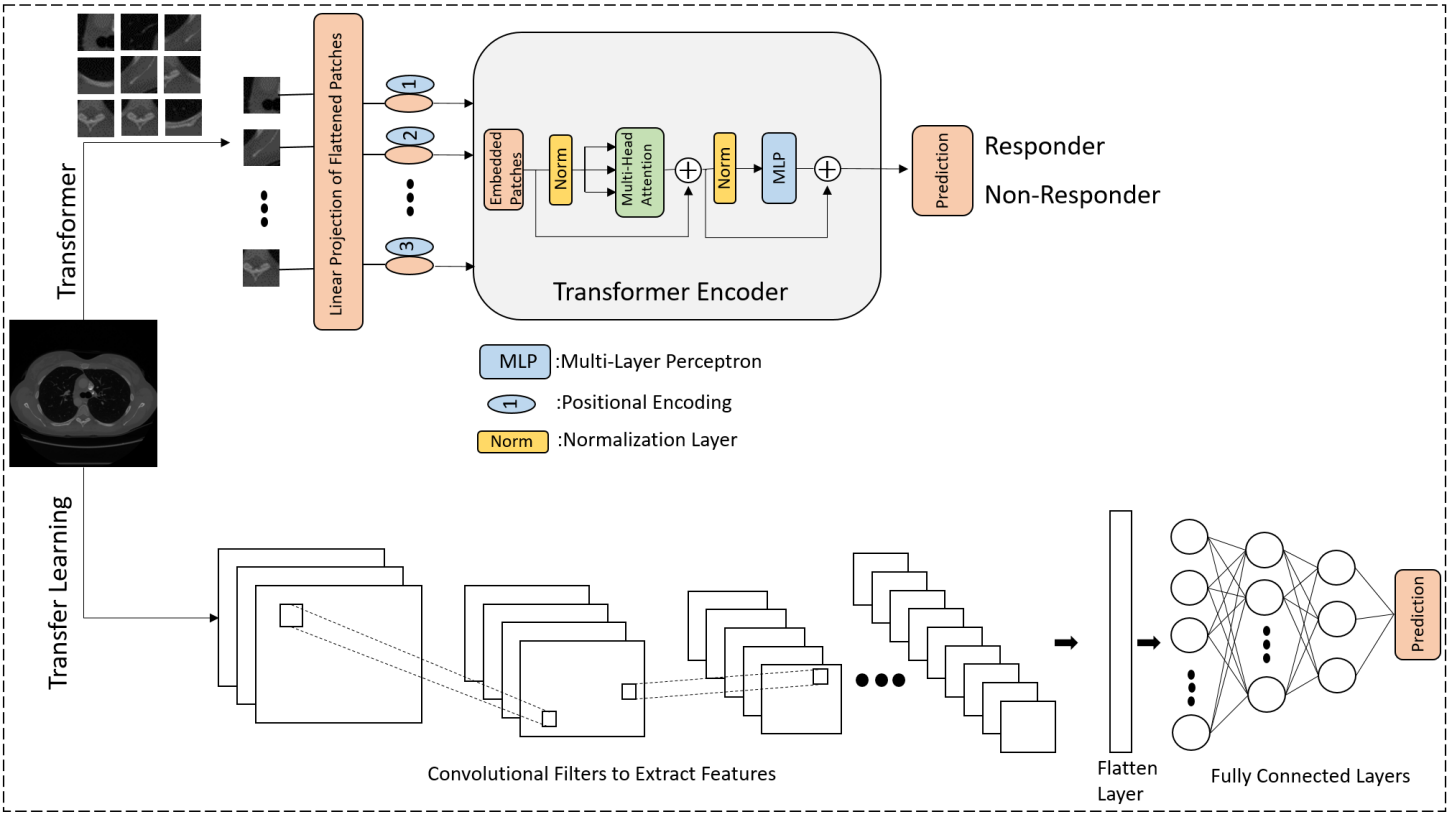
The diagram illustrates a deep learning methodology for forecasting the response to NAC in LABC patients. The lower segment illustrates the application of transfer learning utilizing pre-trained CNNs, while the upper segment illustrates training from the ground up using the Vision Transformer (ViT) approach. In the ViT architecture, images are segmented into patches and converted into a sequential format, akin to the sequence of words in Natural Language Processing (NLP). The positional encoding ensures that each patch’s location retains crucial information. The core component is the transformer encoder, which includes patch embedding transformation, multi-head attention, and MLP.

**Figure 1** shows a schematic of the methods used in the study to predict responder and non-responder patients.

### 2.4 Implementation of deep learning methods

The Python-3 language programing was employed to implement deep-learning methods. Keras 2.11 version was utilized to implement the transformer network and transfer learning networks. Data was split into 60% training set, 10% validation set and 20% test set (70:30 ratio). To see the effect of partitioning percentage on classification accuracy, we tried different train-test ratios including a 75:25 (65% training set, 10% validation set and 25% test set) and a 80:20 (70% training set, 10% validation set and 20% test set) and a 85:15 (75% training set, 10% validation set and 15% test set) and a 90:10 (80% training set, 10% validation set and 10% test set).

Experiments were repeated 10 times (The training and test sets were randomly split ten times to prevent bias towards any particular segment of the dataset.) and the average values of classification performance were reported. For transfer learning, networks were pre-trained on the ImageNet 1k dataset, and ViT was trained from scratch on the available training data.

Data augmentation was implemented using transformations including rotation, translation, zoom and flip. 150 epochs with early stop for training were considered. Learning rate was set to 0.001 and weight decay was set to 0.0001. Dropout rate was set to 0.5, optimizer was “AdamW” and “gelu” was the activation function.

## 3 Evaluation metrics

Accuracy, sensitivity, specificity, and balanced_accuracy of classifications were used to evaluate the performance of classifiers on test data expressed as follows;


$$\begin{array}{c} Accuracy=\frac{TP+TN}{TP+TN+FP+FN}\\ ,\;Sensitivity=\frac{TP}{TP+FN},\;Specificity=\frac{TN}{TN+FP},\;Balanced\_Accuracy=\frac{Sensitivity+Specificity}{2} \end{array}$$

Where TP, TN, FP and FN indicate true positive (true response), true negative (true Non-response), false positive and false negative, respectively.

## 4 Results

In this study, there were 117 women with a mean age of 52 ± 11 (mean ± standard deviation) years. Eighty-two (n=82) participants had a clinical-pathological treatment response (partial or complete response) based on RECIST criteria (37). Thirty-five (n=35) women had no treatment response (stable disease or progressive disease). Invasive ductal carcinoma (IDC) was the major histopathology for patients, and a minority of the patients were diagnosed with invasive lobular carcinoma (ILC) and invasive metaplastic carcinoma (IMC). A majority of patients (42%) had positive estrogen (ER+) and progesterone (PR+) receptors in tumors (major molecular features), and positive Her2/Neu (HER2+) receptor and triple negative tumor (ER-, PR-, HER2) were found in a minority of patients (15% and 22%, respectively). The tumor size changed from 5.2 ± 1.1 cm (mean ± standard deviation) to 1.4 ± 0.4 cm for responders and from 5.6 ± 1.3 cm to 6 ± 1.5 cm in non-responders. Chemotherapy regimens used were doxorubicin (Adriamycin), cyclophosphamide followed by paclitaxel (Taxol) (AC-T), or 5-fluorouracil, epirubicin, cyclophosphamide followed by docetaxel (FEC-D), doxorubicin, cyclophosphamide followed by docetaxel (Taxotere) (AC-D), paclitaxel and cyclophosphamide (TC). Additionally, the monoclonal antibody trastuzumab (Herceptin) (TRA) was utilized for LABC patients with HER2+ tumors. No changes were made to therapy based on imaging in the course of this observational study. **Table 1** provides a summary of the pathological and clinical characteristics of the patients. **Supplementary Table 1** characterizes each patient in terms of their characteristics individually.

**Table 1:** Clinical characteristics of patient cohort.

Characteristics	Responders Mean (std)	Non-responders Mean (std)
**Age**	52 (11)	54 (10)
**Initial Tumour Size**	5.2 (2.5) cm	5.6 (2.7) cm
**Histology**	Percentage (Count)	
IDC	58 (70)	23 (65)
ILC	1 (1)	4 (11)
IMC	3 (3)	2 (5)
**Molecular Features**	Percentage (Count)	
ER+	42 (51)	29 (82)
PR+	37 (45)	24 (68)
HER2+	28 (34)	9 (26)
ER-/PR-/HER2-	22 (27)	4 (11)
ER+/PR+/HER2+	15 (18)	6 (17)
ER+/PR+/HER2-	22 (27)	20 (57)
ER-/PR-/HER2+	15 (18)	4 (11)
**Residual Tumour Size**	1.4 (2.4) cm	6 (5.5) cm
**Response**	Percentage (Count)	
Responding Patients	70 (82)	–
Non-responding Patients	–	30 (35)

std, Standard Deviation; IDC, Invasive Ductal Carcinoma; ILC,Invasive Lobular Carcinoma; IMC,Invasive Metaplastic Carcinoma; ER, estrogen; PR, progesterone.

**Figure 2:**
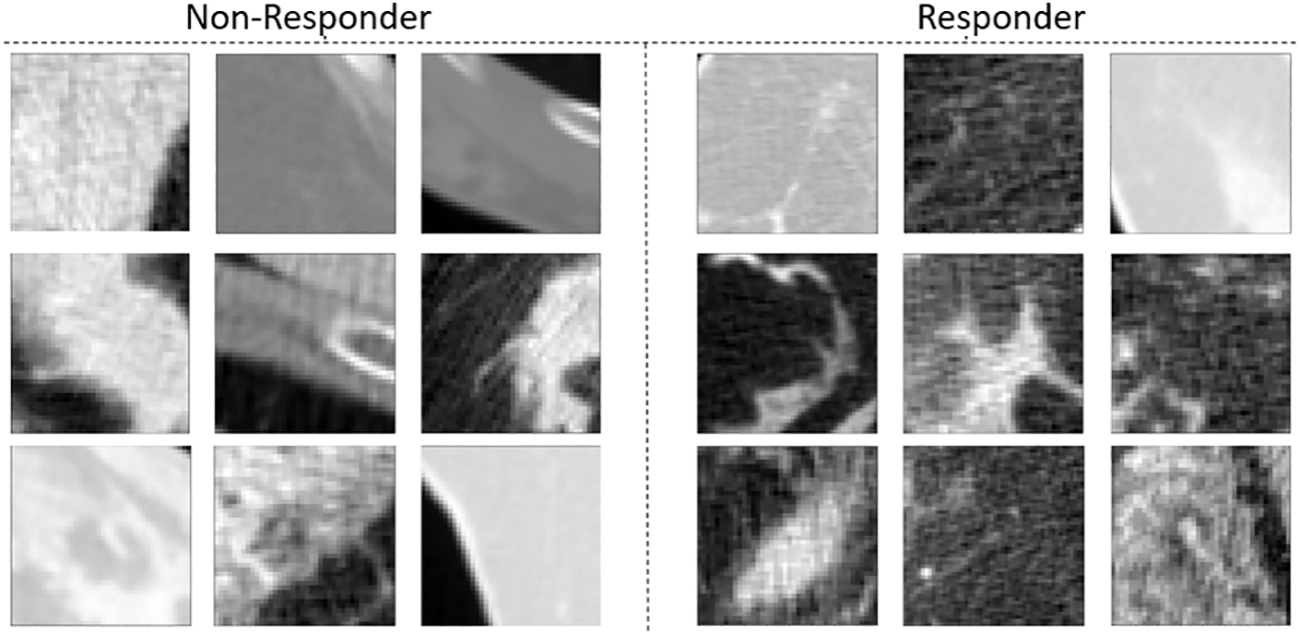
CT images of tumors of patients with LABC who did not respond to treatment (left) and tumors of patients with LABC who did respond to treatment (right).

**Figure 2** presents individual representative CT images from responding and non-responding patients. No apparent differences were visually present.

In terms of response prediction, ViT (Accuracy=77 ± 3, Balanced_Accuracy=69 ± 4) obtained the best performance. Xception with Accuracy=72 ± 4 and Balanced_Accuracy=66 ± 5 placed in second rank, and ResNet-50 obtained third place with Accuracy=72 ± 5 and Balanced_Accuracy=64 ± 4. Results for ViT ranged from accuracy = 71 ± 3% to 77 ± 4%, specificity = 86 ± 6% to 76 ± 3%, sensitivity = 56 ± 4% to 52 ± 4%, and balanced_accuracy=69 ± 3% to =69 ± 3 with different train-test splitting ratios. **Tables 2**–**6** show the performance of networks for different train-test split ratios 90:10, 85:15, 80:20, 75:25 and 70:30, respectively.

**Table 2:** The performance of deep learning networks on the prediction of treatment response for 90:10 ratio (80% train data, 10% validation and 10% test data).

Network \ Metric	Accuracy Mean (SD) %	Balanced_Accuracy Mean (SD) %	Sensitivity Mean (SD) %	Specificity Mean (SD) %
VGG-16	64 (3)	61 (4)	52 (3)	69 (4)
VGG-19	68 (3)	61 (3)	51 (3)	71 (5)
Resnet-50	67 (4)	62 (3)	52 (4)	74 (4)
ResNet-101	61 (5)	63 (4)	52 (3)	70 (4)
Resnet-152	62 (3)	60 (3)	53 (4)	72 (5)
InceptionV3	65 (4)	60 (4)	54 (4)	71 (4)
Xception	67 (4)	62 (5)	53 (3)	75 (4)
Transformer ViT	73 (3)	65 (4)	54 (3)	78 (4)

SD, Standard Deviation

**Table 3:** The performance of deep learning networks on the prediction of treatment response for 85:15 ratio (75% train data, 10% validation and 15% test data).

Network \ Metric	Accuracy Mean (SD) %	Balanced_Accuracy Mean (SD) %	Sensitivity Mean (SD) %	Specificity Mean (SD) %
VGG-16	69 (4)	63 (3)	51 (3)	77 (6)
VGG-19	73 (5)	62 (4)	51 (3)	80 (5)
Resnet-50	72 (5)	64 (4)	53 (4)	82 (6)
ResNet-101	68 (5)	65 (4)	52 (4)	81 (5)
Resnet-152	67 (5)	64 (4)	52 (5)	82 (6)
InceptionV3	69 (6)	63 (5)	51 (3)	82 (5)
Xception	72 (4)	66 (5)	55 (4)	81 (6)
Transformer ViT	77 (3)	69 (4)	56 (4)	86 (6)

SD, Standard Deviation

**Table 4:** The performance of deep learning networks on the prediction of treatment response for 80:20 ratio (70% train data, 10% validation and 20% test data).

Network \ Metric	Accuracy Mean (SD) %	Balanced_Accuracy Mean (SD) %	Sensitivity Mean (SD) %	Specificity Mean (SD) %
VGG-16	63 (4)	58 (4)	50 (4)	67 (4)
VGG-19	66 (4)	60 (4)	50 (3)	70 (4)
Resnet-50	65 (4)	61 (5)	51 (4)	72 (3)
ResNet-101	60 (4)	61 (4)	51 (3)	69 (4)
Resnet-152	61 (4)	58 (4)	51 (4)	71 (4)
InceptionV3	63 (3)	59 (3)	52 (4)	70 (3)
Xception	65 (4)	60 (4)	51 (3)	73 (3)
Transformer ViT	71 (4)	63 (4)	53 (3)	76 (3)

SD, Standard Deviation

**Table 5:** The performance of deep learning networks on the prediction of treatment response for 75:25 ratio (65% train data, 10% validation and 25% test data).

Network \ Metric	Accuracy Mean (SD) %	Balanced_Accuracy Mean (SD) %	Sensitivity Mean (SD) %	Specificity Mean (SD) %
VGG-16	67 (4)	61 (4)	50 (4)	75 (5)
VGG-19	72 (5)	61 (5)	50 (4)	78 (5)
Resnet-50	70 (4)	63 (5)	52 (5)	80 (5)
ResNet-101	65 (4)	64 (5)	50 (4)	79 (4)
Resnet-152	65 (6)	62 (4)	51 (4)	80 (6)
InceptionV3	68 (5)	62 (5)	50 (5)	81 (6)
Xception	70 (5)	64 (6)	53 (5)	80 (5)
Transformer ViT	75 (4)	67 (3)	54 (4)	84 (5)

SD, Standard Deviation

**Table 6:** The performance of deep learning networks on the prediction of treatment response for 70:30 ratio (60% train data, 10% validation and 30% test data).

Network \ Metric	Accuracy Mean (SD) %	Balanced_Accuracy Mean (SD) %	Sensitivity Mean (SD) %	Specificity Mean (SD) %
VGG-16	65 (4)	60 (4)	51 (4)	71 (5)
VGG-19	69 (4)	60 (5)	50 (4)	75 (5)
Resnet-50	68 (4)	61 (5)	51 (5)	78 (5)
ResNet-101	63 (4)	62 (5)	51 (4)	74 (4)
Resnet-152	64 (5)	61 (4)	52 (4)	75 (6)
InceptionV3	67 (5)	61 (5)	52 (5)	74 (6)
Xception	69 (5)	63 (6)	52 (5)	78 (5)
Transformer ViT	74 (5)	66 (3)	52 (4)	81 (5)

SD, Standard Deviation

We applied a t-test to the resulted balanced _accuracy of different networks and this statistical test demonstrated that results are statistically significant.

## 5 Discussion

In this study, two different approaches of deep learning were applied to predict treatment response to NAC for patients with LABC. CT images of 117 patients with LABC were collected prior to the start of NAC treatment for gross disease. Response to NAC treatment was evaluated using standard clinical methodology for ground truth labelling. Specifically, the assessment of the chemotherapy treatment response was determined following the conclusion of the NAC regimen, using standard clinical RECIST criteria as well as histopathological methods.

The ViT technique obtained the best result in comparison with the other transfer learning techniques. This demonstrates that the attention mechanism improved the performance of the algorithm by applying different weights for different parts of an image. The important parts of the image received more attention during the training phase leading to better learning. Additionally, the effect of unimportant parts of the image is considerably decreased, which leads to less redundant information. ViT excels at efficiently capturing global contextual information due to its mechanism. In contrast to CNNs, which depend on local receptive fields and pooling layers, ViT simultaneously analyzes the entire image, enabling it to effectively model extensive dependencies over long ranges (36).

In terms of transfer learning networks, Xception, which is inception with depth-wise separable convolutions, obtained the best performance among all CNN-based networks. Likewise, ResNet50 obtained the second best among all CNN networks. The performance of VGG16 was not promising and it ranked as the last network in terms of classification accuracy. Although VGG16 effectively captures a diverse range of features, it does not explicitly acquire spatial hierarchies. In contrast, contemporary architectures like ResNets have incorporated skip connections and feature reuse mechanisms, enhancing their ability to capture both low-level and high-level features more efficiently.

CT Imaging is not able to visualize the details of cellular structures because of its resolution limitations. However, there might be variations in cellular structure and density, and arrangement which carry significant important information about treatment response. To this end, several studies have demonstrated the correlation between cellular micro-structure characteristics and tumor response (38–40). Additionally, voxel intensity in CT imaging, which shows the attenuation coefficient of tissue, can be used as a good feature to evaluate the variations in tissue micro-structure (41). In order to tackle the challenge of tumor tissue micro-structure characterization using CT, textural features quantification techniques have been frequently employed. To this end, Sadeghi et al. (42) extracted textural features from optical spectroscopic (DOS) images using the grey level co-occurrence matrix (GLCM) technique to predict NAC response in an LABC study. Tran et al. (19) utilized DOS-GLCM textural features to predict NAC response to LABC by training different machine learning classifiers. Tadayyon et al. (20) extracted features from quantitative ultrasound (QUS) to assess the tumor response to NAC for patients with LABC. Dastjerdi et al. (43) combined first-order and second-order GLCM features, which are extracted from CT, to predict the tumor response to NAC.

In other work, Teruel et al. (39) used GLCM features which are extracted from dynamic contrast-enhanced MRI (DCE-MRI) to predict the response of NAC for LABC patients. Cheng et al. (40) applied textural features extracted from 18F-FDG PET/CT images in order to predict pathological complete response (pCR) to NAC. Imaging parameters were maximum standardized uptake value, metabolic tumor volume, and total lesion glycolysis, while textural features included entropy, coarseness, and skewness. They found that variations in textural features after two cycles of treatment could be found in both HER2- and HER2+ patients.

Nevertheless, feature engineering is an essential step for using radiomics features; however, deep learning techniques do not need feature selection. Additionally, in deep learning, detailed features can be extracted by adding more layers. Although adding more layers increases the computational time, as well as the probability of overfitting and gradient vanishing, these challenges can be ameliorated using dropout techniques and regularization constraints. Furthermore, the use of an attention mechanism can increase the weight of important parts of an image, whereas machine learning-based techniques do not have this option. CNN-based deep learning and transformers can be used for end-to-end tasks such as tumor segmentation, feature extraction, and classification using a deep learning network (44). Additionally, the reproducibility of radiomics features is significantly affected by the protocol of feature extraction, which is not a limitation of deep-learning methods.

Jalalifar et al. (23) employed the InceptionResNetV2 network and transformer to extract features from MRI to predict the response of radiotherapy for brain metastasis patients. The transformer was used to preserve spatial dependencies among MRI slices. In another study, Jalalifar et al. (34) proposed a method based on data-efficient image transformer (DEiT) to use ViT for chest X-ray abnormality detection. They considered a teacher-student strategy to train the network such that DensNet is the teacher and ViT is the student. Saednia et al. (31) trained a hierarchical self-attention deep learning network to predict the response of NAC to LABC using digital histopathological images.

The study here demonstrated the potential of employing deep learning networks to predict the response of LABC patients to NAC. The outcomes underscored the efficacy of these networks in terms of both sensitivity and specificity. Furthermore, the study sheds light on the pivotal role of the attention mechanism within the transformer model in enhancing prediction performance. Identifying non-responders to NAC treatment among LABC patients is a formidable challenge, as any deviations from the standard treatment protocol may introduce complications for those patients who do respond. To address this, the study assigned equal importance weights to both non-responders and responders, striking a balance between sensitivity and specificity.

The primary objective of this research was to develop an expert recommender system aimed at optimizing NAC treatment. Physicians could leverage this artificial intelligence-based system to customize treatments and enhance their effectiveness. This system harnessed the power of routine diagnostic CT images and deep learning algorithms to forecast whether a patient would respond to NAC or if an alternative regimen should be considered. A notable limitation of the study was the size of the dataset, which could restrict its generalizability. Since the dataset was small, a considerable difference could not be found in changing the ratio of the training set and test set. Moreover, the validation of results using an external cohort dataset could be instrumental in assessing the technique’s robustness and gauging the algorithm’s applicability beyond the initial dataset. Furthermore, it is worth noting that all patients in the study originated from a single medical center. Although this homogeneity aids in training the algorithm for consistency, incorporating data from multiple centers would enhance the algorithm’s generalizability by accounting for variations associated with diverse practices across different sites. For future work, we can train ViT on large medical image datasets and subsequently fine-tune it on our LABC dataset. Additionally, using generative models such as generative adversarial networks (GAN) or diffusion probabilistic models can improve performance. Particularly, using GAN to augment data in the training phase may improve training.

In summary, this research demonstrated the capacity of deep learning networks, including transformers and transfer learning, to predict the response to NAC treatment in LABC patients before the commencement of treatment. The methodology involved applying various transfer learning networks, such as ViT transformer, VGG16, VGG19, ResNet-50, ResNet-101, ResNet-152, InceptionV3, and Xception, to extract features from CT images for predicting treatment response prior to start. Notably, the ViT transformer exhibited the highest performance, underscoring the effectiveness of the attention mechanism. The results from this preliminary study, particularly the accuracy of predictions, hold promise, indicating that this algorithm can serve as a valuable recommender system for forecasting NAC response before treatment commencement.

## Data availability statement

Data can be made available upon request and review by Institutional Review Board (IRB). Data requests may be sent to Dr. Kullervo Hynynen, Vice-president, Research & Innovation, Sunnybrook Research Institute (khynynen@sri.utoronto.ca).

## Ethics statement

The studies involving humans were approved by Sunnybrook Research Ethics Board. The studies were conducted in accordance with the local legislation and institutional requirements. Written informed consent for participation in this study was provided by the participants’ legal guardians/next of kin.

## Author contributions

AM: Investigation, Methodology, Software, Writing – original draft, Writing – review & editing. LO: Writing – review & editing. AD: Writing – review & editing. DA: Writing – review & editing. MT: Writing – review & editing. SG: Writing – review & editing. AE: Writing – review & editing. FW: Writing – review & editing. NL-H: Writing – review & editing. BC: Writing – review & editing. MK: Writing – review & editing. GJC: Conceptualization, Supervision, Writing – review & editing.
