# Supplementary material for: Apriori prediction of chemotherapy response in locally advanced breast cancer patients using CT imaging and deep learning: transformer versus transfer learning
Source: Front Oncol. 2024 May 2;14:1359148. doi: 10.3389/fonc.2024.1359148 (PMC11096486; doi:10.3389/fonc.2024.1359148)
Supplement: Supplementary file 1 [file Table_1.docx]

**Supplementary Table 1: Patient characteristics and neoadjuvant treatment response results.**

| **No** | **Age** | **Menopausal status** | **Pre-Tx tumour size (cm)** | **Histology** | **Tumour Grade** | **ER/**  **PR/**  **Her2** | **Treatment** | **Post-Tx tumour size** | **Response** |
| --- | --- | --- | --- | --- | --- | --- | --- | --- | --- |
| 1 | 53 | post | 5.4 | IDC | I | --+ | FECD + TRA | 0.0 | R |
| 2 | 31 | pre | 5.0 | IDC | I | ++- | ACT | 1.4 | R |
| 3 | 46 | pre | 8.0 | IDC | III | --- | ACT | 6.4 | NR |
| 4 | 39 | pre | 10.0 | IDC | II | +++- | FECD | 8.0 | R |
| 5 | 43 | post | 8.0 | IDC | II | +++ | ACT+TRA | 0.0 | R |
| 6 | 48 | pre | 4.9 | IDC | III | ++- | ACT | 1.4 | R |
| 7 | 36 | pre | 5.8 | IDC | III | +++ | ACT | 11.4 | NR |
| 8 | 40 | post | 4.4 | IDC | III | --- | ACT | 0.0 | R |
| 9 | 59 | post | 6.0 | IDC | III | --+ | ACT | 2.6 | R |
| 10 | 65 | pre | 6.7 | IDC | II | +-- | ACT+TRA | 5.5 | NR |
| 11 | 38 | pre | 9.2 | IDC | II | ++- | ACT | 4.5 | R |
| 12 | 53 | pre | 11.7 | IMC | III | --- | ACT | 9.4 | NR |
| 13 | 48 | pre | 9.0 | IDC | II | +++ | ACT | 5.0 | R |
| 14 | 49 | pre | 5.6 | IDC | II | --+ | ACT+TRA | 0.1 | R |

**Supplementary Table 1 continue…**

| 15 | 47 | post | 5.2 | IDC | II | ++- | FECD | 6.5 | R |
| --- | --- | --- | --- | --- | --- | --- | --- | --- | --- |
| 16 | 38 | pre | 8.0 | IDC | III | --+ | ACT | 0.0 | R |
| 17 | 47 | post | 9.9 | IDC | II | ++- | ACT | 18.0 | NR |
| 18 | 57 | post | 5.5 | IDC | III | --- | ACT | 0.0 | R |
| 19 | 59 | peri | 4.7 | IDC | N/A | ++- | ACT | 2.1 | R |
| 20 | 47 | pre | 7.4 | IDC | N/A | --+ | ACT+TRA | 0.0 | R |
| 21 | 55 | pre | 12.8 | IDC | II | ++- | ACT | 17.0 | NR |
| 22 | 62 | post | 10.0 | IDC | III | +++ | FECD | 7.0 | R |
| 23 | 32 | pre | 7.0 | IMC | N/A | +++ | ACT+TRA | 7.4 | R |
| 24 | 38 | pre | 2.5 | IDC | III | --- | ACT | 3.8 | NR |
| 25 | 45 | pre | 6.0 | IDC | I | +++ | ACT+TRA | 4.8 | NR |
| 26 | 55 | post | 10.5 | IDC | III | --- | ACT | 0.1 | R |
| 27 | 59 | post | 8.0 | IDC | II | +-+ | FECD+TRA | 0.0 | R |
| 28 | 45 | pre | 3.8 | IDC | II | ++- | ACT | 0.5 | R |
| 29 | 37 | pre | 3.6 | IDC | III | ++- | ACTA | 2.2 | R |
| 30 | 50 | pre | 9.0 | IDC | II | +++ | ACT+TRA | 1.2 | R |
| 31 | 54 | peri | 3.6 | IDC | N/A | ++- | DC | 1.7 | R |
| 32 | 55 | pre | 1.6 | IMC | I | +-- | DC | 1.2 | NR |
| 33 | 50 | post | 7.3 | IDC | III | --- | FECD | 2.1 | R |
| 34 | 55 | post | 3.4 | IDC | III | --- | ACT | 1.8 | R |
| 35 | 32 | post | 2.7 | IDC | II | +-+ | ACT+TRA | 0.1 | R |
| 36 | 64 | post | 8.7 | ILC | II | ++- | FECD | 19.0 | NR |
| 37 | 67 | post | 2.5 | IDC | II | --- | FECD | 3.2 | R |
| 38 | 52 | post | 2.6 | IDC | II | --- | FECD | 2.5 | R |

**Supplementary Table 1 continue…**

| 39 | 56 | pre | 7.0 | IDC | II | +++ | ACT+TRA | 8.4 | NR |
| --- | --- | --- | --- | --- | --- | --- | --- | --- | --- |
| 40 | 45 | post | 2.3 | IDC | N/A | +++ | FECD+TRA | 0.0 | R |
| 41 | 59 | post | 4.9 | IDC | II | ++- | FECD | 2.8 | NR |
| 42 | 67 | pre | 7.4 | IDC | III | ++- | FECD | 3.3 | R |
| 43 | 49 | pre | 2.1 | IDC | II | +-+ | ACT+TRA | 0.0 | R |
| 44 | 62 | pre | 6.3 | IDC | II | --- | ACT | 12.6 | NR |
| 45 | 58 | post | 5.2 | IDC | I | +++ | ACT+TRA | 3.4 | R |
| 46 | 58 | pre | 4.0 | IMC | III | --+ | DCB+TRA | 0.0 | R |
| 47 | 45 | pre | 4.0 | IDC | II | ++- | ACT | 3.0 | NR |
| 48 | 29 | pre | 4.2 | IDC | III | ++- | ACT | 4.0 | NR |
| 49 | 79 | post | 3.9 | IDC | II | --+ | ACT+TRA | 0.1 | R |
| 50 | 42 | pre | 9.6 | IDC | N/A | ++- | FECD | 3.0 | R |
| 51 | 66 | post | 3.0 | IDC | III | +-+ | FECD+TRA | 2.4 | NR |
| 52 | 38 | pre | 5.0 | IDC | III | --- | ACT | 5.0 | NR |
| 53 | 40 | pre | 11.7 | IDC | III | +++ | ACT+TRA | 1.3 | R |
| 54 | 53 | post | 8.8 | IDC | II | --- | FECD | 2.5 | R |
| 55 | 47 | pre | 3.5 | IDC | II | ++- | ACT | 4.0 | NR |
| 56 | 57 | peri | 3.9 | IDC | II | +-- | ACT | 3.3 | NR |
| 57 | 53 | post | 5.6 | IDC | III | --+ | ACT+TRA | 0.2 | R |
| 58 | 55 | post | 7.9 | IDC | II | ++- | ACT | 12.6 | R |

**Supplementary Table 1 continue…**

| 59 | 51 | pre | 2.2 | IDC | N/A | ++- | ACT | 0.5 | R |
| --- | --- | --- | --- | --- | --- | --- | --- | --- | --- |
| 60 | 38 | pre | 10.8 | IDC | II | ++- | FECD | 4.9 | R |
| 61 | 72 | post | 3.3 | IDC | II | +-- | ACT | 0.2 | R |
| 62 | 41 | pre | 4.5 | IDC | III | --- | ACT | 2.0 | R |
| 63 | 48 | pre | 5.6 | ILC | II | ++- | FECD | 11.0 | NR |
| 64 | 83 | post | 7.7 | IDC | III | +-+ | ACT+TRA | 0.0 | R |
| 65 | 51 | post | 4.5 | IDC | III | ++- | ACT | 3.5 | NR |
| 66 | 43 | pre | 9.0 | IDC | III | --- | ACT | 3.9 | R |
| 67 | 42 | pre | 5.0 | IMC | III | ++- | FECD | 8.0 | NR |
| 68 | 60 | post | 7.2 | IDC | III | +++ | ACT+TRA | 1.0 | R |
| 69 | 42 | pre | 6.3 | IDC | II | ++- | FECD | 8.4 | NR |
| 70 | 42 | pre | 4.1 | IDC | I | ++- | ACT | 4.5 | NR |
| 71 | 47 | pre | 10.4 | IDC | II | ++- | ACT | 4.5 | R |
| 72 | 45 | pre | 7.3 | IDC | II | ++- | FECD | 1.4 | R |
| 73 | 43 | pre | 8.5 | IMC | II | --- | FECD | 0.01 | R |
| 74 | 45 | pre | 6.2 | IDC | II | +++ | FECD+TRA | 4.0 | R |
| 75 | 50 | pre | 4.2 | IDC | II | +++ | FECD+TRA | 1.9 | R |
| 76 | 62 | post | 2.1 | IDC | I | --- | DCB | 0.0 | R |
| 77 | 69 | post | 7.3 | ILC | II | +++ | ACT+TRA | 0.0 | R |

**Supplementary Table 1 continue…**

| 78 | 42 | pre | 6.0 | IDC | II | +++ | FECD+TRA | 2.5 | R |
| --- | --- | --- | --- | --- | --- | --- | --- | --- | --- |
| 79 | 72 | pre | 1.3 | IDC | II | +++ | TC+TRA | 0.0 | R |
| 80 | 70 | post | 5.8 | IDC | I | ++- | FECD | 14.4 | NR |
| 81 | 45 | pre | 2.0 | IDC | I | ++- | FECD | 0.2 | R |
| 82 | 42 | pre | 3.8 | IDC | III | ++- | ACT | 1.0 | R |
| 83 | 63 | post | 8.6 | IDC | II | ++- | ACT | 7.5 | NR |
| 84 | 52 | pre | 3.1 | IDC | II | --- | FECD | 0.2 | R |
| 85 | 61 | post | 8.2 | IDC | II | ++- | ACT | 3.0 | NR |
| 86 | 54 | post | 2.3 | IDC | III | --- | FECD | 0.0 | R |
| 87 | 51 | post | 3.5 | IDC | III | +++ | ACT+TRA | 1.2 | R |
| 88 | 45 | post | 4.3 | IMC | II | ++- | FECD | 1.9 | R |
| 89 | 45 | post | 3.2 | IMC | III | --- | ACT | 3.3 | NR |
| 90 | 56 | post | 2.9 | ILC | III | ++- | FECD | 2.2 | NR |
| 91 | 42 | post | 2.1 | IDC | III | +++ | FECD+TRA | 0.0 | R |
| 92 | 38 | post | 1.9 | IDC | III | --+ | ACT+TRA | 0.8 | R |
| 93 | 36 | post | 2.9 | IDC | III | +++ | FECD+TRA | 0.1 | R |
| 94 | 27 | post | 11.3 | IDC | II | +++ | ACT+TRA | 2.5 | R |
| 95 | 36 | post | 5.1 | IDC | III | --- | ACT | 2.5 | R |
| 96 | 66 | post | 0.8 | IDC | I | ++- | ACT | 0.0 | R |
| 97 | 53 | post | 3.9 | IDC | N/A | ++- | ED | 3.7 | NR |
| 98 | 62 | post | 3.5 | IDC | I | ++- | FECD | 0.0 | R |

**Supplementary Table 1 continue…**

| 99 | 63 | post | 3.7 | IDC | III | --- | ACT | 0.0 | R |
| --- | --- | --- | --- | --- | --- | --- | --- | --- | --- |
| 100 | 81 | post | 3.8 | IDC | III | --- | DC | 9.0 | NR |
| 101 | 55 | post | 3.4 | IMC | III | --- | ACT | 1.5 | R |
| 102 | 47 | post | 3.0 | IDC | III | +-- | ACT | 2.7 | NR |
| 103 | 34 | post | 1.9 | IDC | III | --- | DC | 1.3 | R |
| 104 | 67 | post | 1.9 | IDC | III | --- | ACT | 0.0 | R |
| 105 | 51 | peri | 1.9 | IDC | III | --- | ACT | 2.5 | NR |
| 106 | 37 | peri | 4.4 | IDC | III | ++- | FECD | 2.5 | R |
| 107 | 45 | peri | 2.7 | IDC | III | --+ | ACT+TRA | 2.5 | R |
| 108 | 49 | peri | 2.5 | IDC | III | +++ | ACT+TRA | 2.5 | NR |
| 109 | 50 | peri | 10.7 | IDC | II | +++ | ACT+TRA | 2.4 | R |
| 110 | 51 | peri | 4.0 | IDC | III | ++- | FECD | 2.0 | R |
| 111 | 40 | peri | 5.9 | IDC | II | ++- | FECD | 6.0 | R |
| 112 | 72 | post | 3.5 | IDC | III | +++ | FECD+TRA | 0.0 | R |
| 113 | 51 | peri | 3.3 | IDC | III | ++- | ACT | 5.5 | R |
| 114 | 63 | post | 4.0 | IDC | III | --- | ACT | 0.5 | R |
| 115 | 56 | post | 3.5 | IDC | III | +-- | DC | 1.9 | R |

**Supplementary Table 1 continue…**

| 116 | 49 | pre | 4.9 | IDC | II | +++ | FECD+TRA | 2.5 | NR |
| --- | --- | --- | --- | --- | --- | --- | --- | --- | --- |
| 117 | 50 | pre | 3.0 | IDC | III | ++- | ACT | 3.2 | R |

ILC: invasive lobular carcinoma, IDC: invasive ductal carcinoma, IMC: invasive micropapillary carcinoma, N/A: not available, ACT: Adriamycin and Cytoxan + Taxotere, FECD: Fluorouracil, epirubicin and cyclophosphamide + docetaxel, TC: Taxotere and cyclophosphamide, TRA: trastuzumab, D: docetaxel, XRT: radiation treatment, DC: docetaxel and cyclophosphamide, DCB: docetaxel and carboplatin, ED: epirubicin and docetaxel, CIS: cisplatin, R: Responder, and NR: Non-responder.
